# MEDUSA: Marine benthic Ecological Data from Underwater imagery Surveys of sub-Antarctic Crozet environments

**DOI:** 10.1038/s41597-024-03460-4

**Published:** 2024-06-12

**Authors:** Yann Lelièvre, Sébastien Motreuil, Léa Specq, Christian Marschal, Philippe Dubois, Lisa Wauters, Mathilde Guéné, Thomas Saucède

**Affiliations:** 1 Biogéosciences, UMR 6282 CNRS, Université de Bourgogne, 6 Boulevard Gabriel, 21000 Dijon, France; 2 IMBE-Institut Méditerranéen de Biologie et d’Ecologie marine et continentale, UMR 7263 Aix Marseille Université/CNRS/IRD/UAPV, Station Marine d’Endoume, Chemin de la Batterie des Lions, 13007 Marseille, France; 3 Laboratoire de Biologie marine, Université Libre de Bruxelles, Avenue F.D. Roosevelt 50, CP160/15, 1050 Bruxelles, Belgium; 4 Direction de l’Environnement, Terres australes et antarctiques françaises, Rue Gabriel Dejean, 97410 Saint-Pierre, La Réunion France

**Keywords:** Biodiversity, Ecosystem ecology, Conservation biology, Marine biology, Environmental impact

## Abstract

Inscribed on the UNESCO World Heritage list, the sub-Antarctic Crozet archipelago is located in a region facing significant environmental changes impacting a poorly known marine biodiversity. Underwater imagery constitutes a valuable non-invasive approach for gathering ecological data and improving our knowledge of ecosystems’ vulnerability. We here compiled two datasets, encompassing 17 video-imagery surveys of Crozet nearshore environments conducted in 2021 and 2022 at two sites of *Ile de la Possession*: *Baie du Marin* and *Crique du Sphinx*. Faunal abundance and algal cover data related to each survey are also provided. A total of 755 images were analysed, comprising 52 faunal and 14 algal taxa identified in 2021, as well as 45 faunal and 14 algal taxa identified in 2022. Video-transects were performed in shallow waters by scuba divers using a GoPro^®^HERO7 multiple camera set-up, and in deeper waters using a remotely operated vehicle. These data provide a first baseline for biodiversity and ecosystem studies, and for monitoring the long-term dynamics of Crozet benthic habitats facing natural and anthropogenic disturbances.

## Background & Summary

Atmospheric temperature records have clearly indicated a long-term warming trend in the Southern Ocean over the twentieth and beginning of the twenty-first centuries^1–6^, including the French sub-Antarctic islands such as the Crozet archipelago. At Crozet, air temperature increase has been estimated to be +1.2 °C/100 years^5^. This global warming is coupled with underlying phenomena, such as significant seawater temperature increase^7,8^, ocean acidification^9^, extreme climatic events and pronounced seasonality^10,11^. These environmental changes are expected to induce substantial alterations of the structure and functioning of sub-Antarctic marine ecosystems^12^, as already observed at Prince Edward Islands^13^. A good knowledge of these marine environments is a prerequisite to understand the impacts of global change and to establish sustainable management policies.

Coastal habitats are highly productive areas inhabited by dense marine vegetation and a diverse array of benthic organisms^14^. They exhibit important variations of structural complexity, substrate composition, and environmental conditions, leading to a large diversity of ecological niches that influence the distribution, composition, and structure of benthic communities^15–17^. Faunal^12,18–22^ and algal^12,23^ diversities are unique in sub-Antarctic nearshore marine ecosystems. However, the slow growth rate of benthic organisms makes them particularly at risk with regards to environmental disturbances^24–26^. As also reported along the Antarctic Peninsula^27^, Lelièvre *et al*.^12^ highlighted that faunal communities of *Ile de la Possession* (Crozet archipelago) displayed functional characteristics indicating a potential vulnerability to diversity loss due to current and future environmental changes. These findings emphasize the importance to conduct diversity studies to track the long-term evolution of these communities and evaluate their resilience to environmental changes.

Conducting benthic studies in these isolated territories is challenging considering extreme environmental conditions (e.g., sea and meteorological conditions), logistics issues and financial limitations. Whereas a thorough knowledge of the biodiversity and ecological functioning of these polar ecosystems is necessary for their protection and conservation, the complex interplay between these multiple limitations hampers the acquisition of ecological data. Imaging technologies stand as powerful and suitable approaches to enhance our understanding of marine habitats^12,18,28–30^. Ethical considerations underscore the value of underwater video-monitoring methods as they provide a non-invasive means to gather highly informative data^29^. By covering large surfaces and providing repeatable measurements^29,30^, video-imagery can give an extended overview of habitats and benthic communities, and constitutes an important tool for environment monitoring and evaluation of the potential impacts of climate change and anthropogenic activities on sub-Antarctic ecosystems^12^.

Although the maritime domain surrounding Crozet Islands is managed as a nature reserve and was classified on the UNESCO World Heritage list in 2019, datasets on benthic ecosystems are still limited^12^. In the present data paper, we describe video-imagery datasets acquired at Crozet in 2021 and 2022 as an outcome of project No. 2021-0882 “Nearshore Cable Inspection and Environmental Survey at IMS Hydroacoustic Station HA04 Crozet, France” conducted by the French Southern and Antarctic Territories (TAAF) in the frame of the Comprehensive Nuclear-Test-Ban Treaty Organization (CTBTO)^31^ partnership, and to the French Polar Institute project #1044 Proteker. Imagery datasets were collected by using an image acquisition set-up in shallow waters (from 0 to 20 m depth) and a remotely operated vehicle (ROV) in deeper waters (from 20 to 60 m depth). The MEDUSA (Marine benthic Ecological Data from Underwater imagery Surveys of sub-Antarctic Crozet environments) dataset includes (i) 17 video-transects recorded at two sites along the eastern coast of *Ile de la Possession*, *Baie du Marin* and *Crique du Sphinx*; as well as (ii) biological data on benthic communities (faunal abundance and algal surface cover) obtained from the analysis of the video-transect imagery. To our knowledge, these still images and video-datasets are the very first records of benthic ecosystems conducted at the Crozet archipelago, offering to users a wide range of imaging processing and analysis possibilities. The MEDUSA dataset brings novel and valuable information to our understanding of the structure and functioning of Crozet benthic nearshore marine ecosystems, and opens new research avenues for sub-Antarctic ecological studies in general.

## Methods

### Study areas

**Fig. 1:**
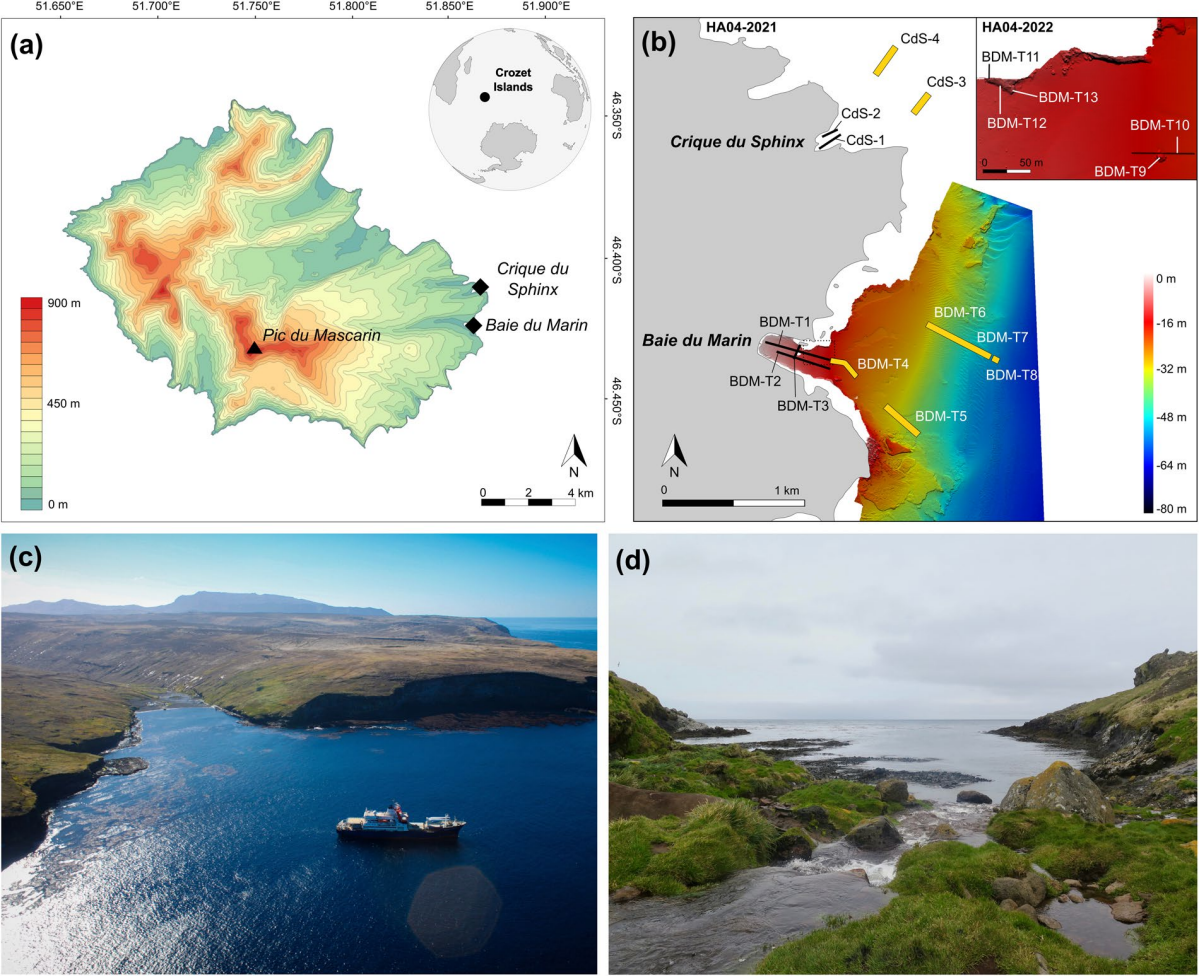
(**a**) Topographic map of *Ile de la Possession* (Crozet archipelago, Southern Ocean) with the location of both study sites; (**b**) Overview of the video transect surveys’ sampling design conducted at *Baie du Marin* and *Crique du Sphinx* in 2021 and 2022. More details on the HA04-Crozet-2022 imagery sampling design are provided in Lelièvre *et al*.^12^. Bathymetry product of CTBTO-IPEV-TAAF cruise MD202/CTBTO-CRO *R/V Marion Dufresne*, Mesuris survey (2016); Photographs of (**c**) *Baie du Marin*. Photo courtesy of *Comprehensive Nuclear-Test-Ban Treaty Organization*; and (**d**) *Crique du Sphinx*. Photo courtesy of Proteker project.

Discovered in 1772 during the expedition of the French explorer Marc Joseph Marion-Dufresne, the Crozet archipelago (45°48′S – 46°26′S; 50°14′E – 52°15′E) – located at 2,400 km north of the Antarctic Continent and 2,400 km southeast of South Africa – is composed of five small volcanic islands. These islands include, from west to east: *Île aux Cochons*, *Îlots des Apôtres*, *Île des Pingouins*, and the two largest islands of the archipelago, *Île de la Possession and Île de l’Est*^32^. *Ile de la Possession* (46°25′S; 51°45′E; Fig. 1a) – approximately 18 km wide by 15 km long, with a total surface area of ~156 km^2^ – is the emerged part of a stratovolcano reaching an elevation of 934 m above sea level (a.s.l.) (*Pic du Mascarin*). Data presented here were acquired at two sites located on the eastern coast of *Ile de la Possession*, at *Baie du Marin* and *Crique du Sphinx* (Fig. 1b).

*Baie du Marin* (BDM; 46°25′54″S; 51°52′11″E; Fig. 1c) is a narrow inlet of c. 500 m length and 200 m width in its shallowest part (<20 m depth), and opens to the ocean in a larger embayment of about 2 km wide at 40 m depth. The coast is characterised by a rocky shore with a sandy beach located at the back of the bay. The site is distinguished by an important colony of over 10,000 king penguins *Aptenodytes patagonicus* (Miller, 1778) and elephant seals *Mirounga leonina* (Linnaeus, 1758), and harbours a rich benthic faunal and algal diversity^12^. *Crique du Sphinx* (CdS; 46°25′08″S; 51°52′44″E; Fig. 1d) is located at 2 km to the north of *Baie du Marin* and opens northeastward as a small cove, spanning approximately 250 m long by 150 m width. Similarly at *Baie du Marin*, the coast is bordered by a rocky shore, with however a small beach of pebbles and gravels.

### Project background

The site of *Baie du Marin* is host to a hydroacoustic monitoring station (HA04) set up in December 2016 by the Preparatory Commission for the Comprehensive Nuclear-Test-Ban Treaty Organization (CTBTO)^31^. Prior to the HA04 setting up, the Polar Environment Committee, which is consulted for every matter of potential environmental relevance in the French Southern and Antarctic Territories (TAAF), recommended that an environmental survey was conducted and a baseline established for monitoring the impact of the station setting up on marine habitats. TAAF awarded the Commission the “*concession for the utilization of the public maritime domain outside ports for the re−establishment of hydroacoustic station HA04 in the Crozet archipelago in the French Austral and Antarctic Territories*” and required that the Commission be committed to support accompanying conservation measures. TAAF and CTBTO made an agreement for “Nearshore Cable Inspection and Environmental Survey at IMS Hydroacoustic Station HA04 Crozet, France” (Contract No. 2021-0882) in September 2021. The first campaign took place in November 2021 for inspection of the HA04 station cable and environmental surveys of benthic nearshore habitats; and a second campaign was carried out in November 2022.

### Video-imagery set-ups

**Fig. 2:**
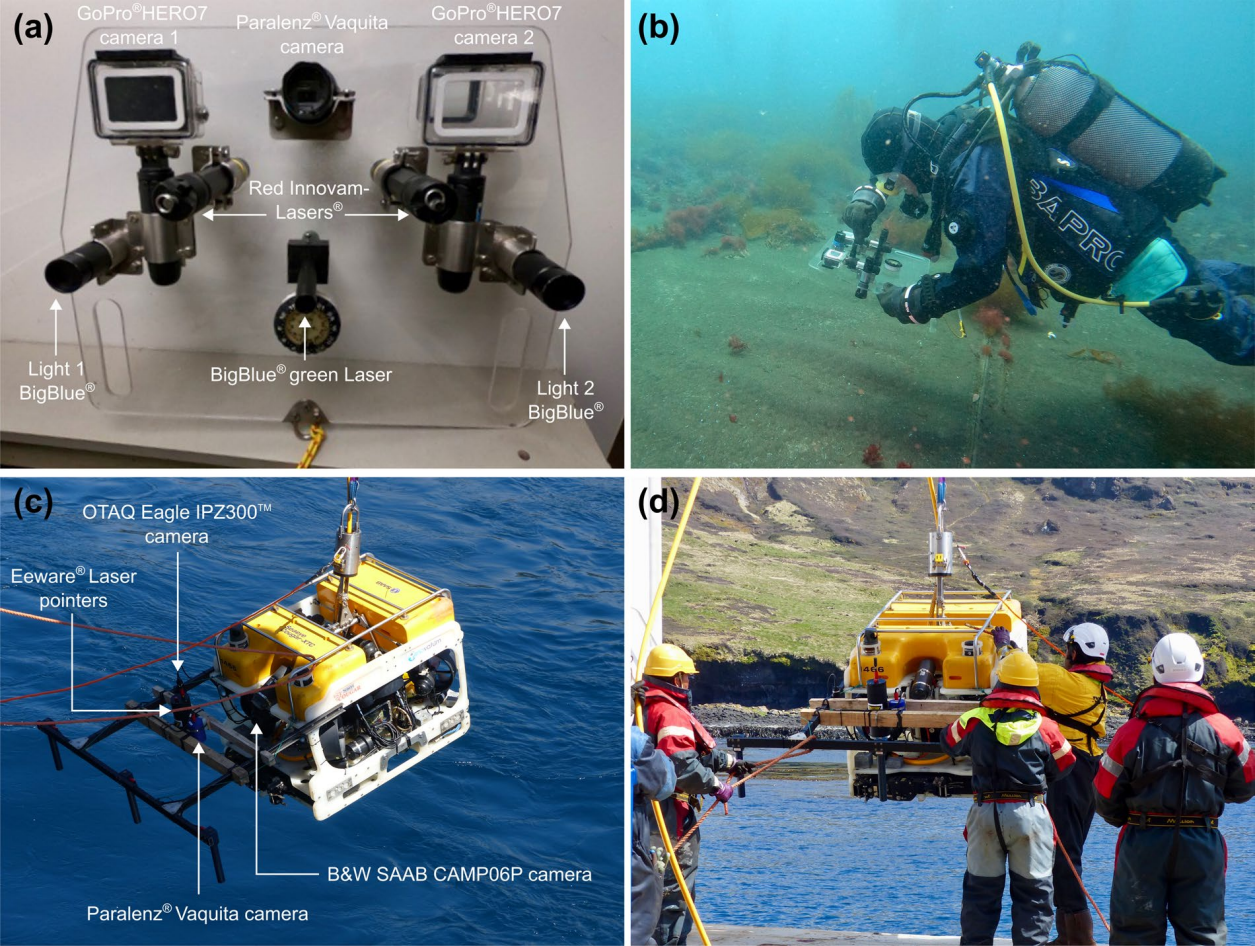
Image acquisition set-ups with the (**a,b**) GoPro^®^HERO7 cameras set-up; and the (**c,d**) remotely operated vehicle *SAAB Seaeye Cougar-XT Compact*.

Benthic video surveys conducted in shallow waters (from 0 to 20 m depth) were performed using a multiple camera set-up (Fig. 2a,b) made of two GoPro^®^HERO7 cameras with 80% field overlap, coupled with a Paralenz^®^ Vaquita 2nd generation camera, all cameras being embedded in an epoxy resin plate. Both GoPro^®^HERO7 cameras were waterproof with Front liquid-crystal display (LCD), 4 K video and 12-megapixel photos resolution (3840 × 2160 p), live streaming stabilization and a 128 GB card. The two GoPro^®^HERO7 cameras were coupled with a waterproof underwater camera Paralenz^®^ Vaquita 2nd generation (video output: 4K-30 fps; power sources: rechargeable Li-Po – 1600 mAh) equipped with depth and temperature sensors. The lighting system consist of two high performance BigBlue^®^ AL1300XWP lights (beam angle: 120°, 1300 lm; cold white colour (6500 K); power sources: rechargeable Li-Ion 18650 battery) placed next to each GoPro^®^HERO7 camera. The set-up was also equipped with three laser beams, two Innovam-Lasers^®^ RLP-LG05-B150 red colour pointers (output power: <5 mW; wavelength: λ = 635 nm; power sources: 2 × AA alkaline) and one BigBlue® green laser pointer (output power: <1 mW; wavelength: λ = 520 nm; power sources: 2 × AAA alkaline). Red lasers were 10 cm apart in 2021, 15 cm in 2022 in order to estimate the filmed surface area. Video surveys were performed by scuba divers who maintained a regular swimming velocity, with the camera oriented parallel to the sea bottom at an average distance of 50 cm.

In deeper waters (from 20 to 60 m depth), video surveys were conducted using the Remotely Operated Vehicle (ROV) *SAAB Seaeye Cougar-XT Compact* (Fig. 2c,d). The ROV was equipped with a colour high-resolution OTAQ Eagle IPZ300™ camera (maximum resolution 1920 × 1080 (1080 P, 25 fps); 18x optical zoom) set perpendicular to the seabed, and an oblique high-resolution black and white SAAB CAM06P rotating front camera. An Eeware® dimensional laser pointer set-up positioned perpendicular to the seabed and forming an equilateral triangle with summits 7 cm apart was employed to estimate the filmed surface area.

### Underwater imagery collection

Image acquisition was performed during *R/V Marion Dufresne* rotations OP03-2021 and OP03-2022. Operations were led by TAAF in response to contract No. 2021-0882 agreed with CTBTO for “Nearshore Cable Inspection and Environmental Survey at IMS Hydroacoustic Station HA04 Crozet, France”, from 4^th^ November to 9^th^ November 2021, and from 23^rd^ November to 24^th^ November 2022, respectively.

Throughout the HA04-Crozet-2021 campaign, twelve surveys were performed: eight at *Baie du Marin* and four at *Crique du Sphinx*. At *Baie du Marin*, a first transect (BDM-T1) was performed along the submarine cables. A second one (BDM-T2) was done 10 m apart to the south, and a third one (BDM-T3) was done across the bay starting from the center of the bay, at 10 m depth in direction of the northern coast. In deeper waters, two ROV surveys were conducted along the submarine cables (BDM-T4 and BDM-T5, respectively), and two surveys in the northern part of the bay (BDM-T6 and BDM-T7). Finally, an additional ROV survey was conducted at 60 m depth (BDM-T8). At *Crique du Sphinx*, two transects (CdS-T1 and CdS-T2) were performed by scuba divers along the cove; and two ROV surveys were performed in the south (CdS-T3) and north (CdS-T4) of the cove.

During the HA04-Crozet-2022 campaign, five video surveys were performed and focused on hard substrates (rocky bottoms and submarine cables) in *Baie du Marin* shallow waters. The first transect (BDM-T9) aimed to survey benthic communities located on the rock close to the submarine cables, at 19 m depth. The second transect (BDM-T10) focused on the suspended section of submarine cables (as conducted also in the BDM-T1) to assess the potential influence of cables as substrate for benthic communities. The following three transects (BDM-T11, BDM-T12 and BDM-T13) were carried out along the rocky shore in the north of the bay, approximately 200 m apart from submarine cables. Benthic communities’ data on hard substrates in 2022 was completed by the image analysis of two transect sections conducted in 2021, encompassing BDM-T1x which represents the suspended-cables section of the transect BDM-T1, and BDM-T3x which includes the rocky substrate from the BDM-T3 transect. Detailed information can be found in Lelièvre *et al*.^12^.

Imagery surveys were conducted in accordance with regulation rules of the French Southern and Antarctic Territories (TAAF) under permits A-2021-98 and A-2022-90 for field access, for HA04-Crozet-2021 and HA04-Crozet-2022 oceanographic campaigns, respectively.

## Data Records

To facilitate data download, each survey of the both oceanographic campaigns conducted at Crozet (HA04-Crozet-2021 and HA04-Crozet-2022) has been individually deposited and is accessible on the dat@UBFC repository (10.25666/DATAUBFC-2024-03-15)^33^. Detailed information regarding each transect are provided in the metadata table deposited on the dat@UBFC repository. Survey folders include two main subfolders corresponding to (i) videos imagery metadata (MOVI: Manually and remotely Operated VIdeo surveys); and (ii) images and benthic communities data (CROCO: CROzet COmmunities data and images).

### MOVI: Manually and remotely Operated VIdeo surveys

**Fig. 3:**
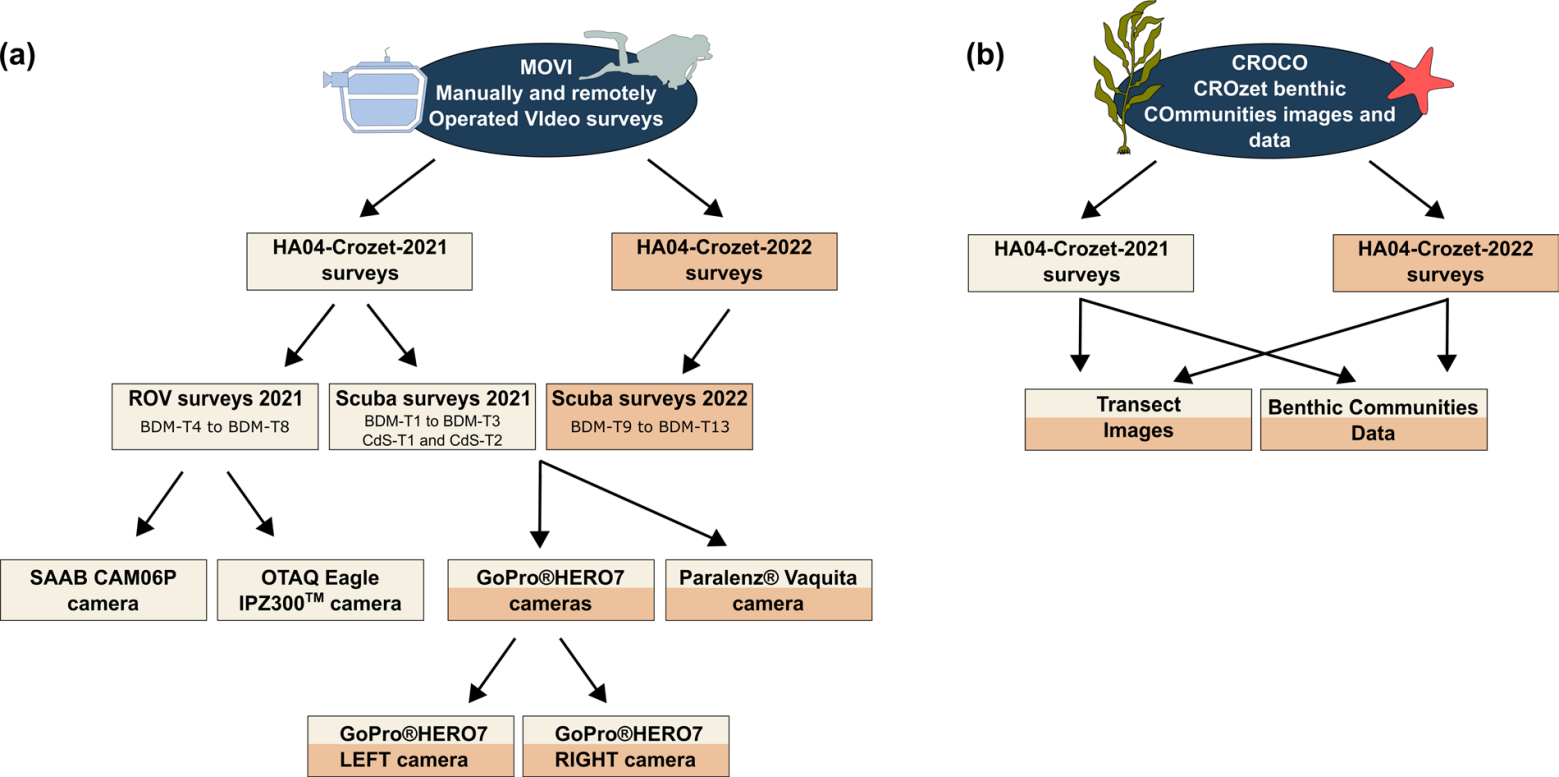
Guide to folders hierarchical structure of the MEDUSA dataset, including (**a**) MOVI (Manually and remotely Operated VIdeo surveys) and (**b**) CROCO (CROzet benthic COmmunities images and data).

For imagery transects conducted during the HA04-Crozet-2021 campaign, scuba surveys (BDM-T1 to BDM-T3; CdS-T1 and CdS-T2) folders includes two additional folders, GoPro^®^HERO7 (further divided into GoPro^®^HERO7 LEFT camera and GoPro^®^HERO7 RIGHT camera) and Paralenz^®^ Vaquita; and surveys carried out using the ROV (BDM-T4 to BDM-T8; CdS-T3 and CdS-T4) includes two folders with video transects of the OTAQ Eagle IPZ300™ camera and of the B&W SAAB CAM06P camera (Fig. 3a). However, surveys collected during the HA04-Crozet-2022 campaign (BDM-T9 to BDM-T13) contains two subfolders: GoPro^®^HERO7 (further divided into GoPro^®^HERO7 LEFT camera and GoPro^®^HERO7 RIGHT camera) and Paralenz^®^ Vaquita (Fig. 3a).

Video surveys in the archives have the following format:

<site-survey ID>_<camera ID-sequence ID>_<year>.<format>

All video surveys have a filename starting with the string: <site-survey ID>, where site name is either BDM (standing for *Baie du Marin*) or CdS (standing for *Crique du Sphinx*), and survey ID starts with letter T for “Transect” followed by a figure comprised between 1 and 13 for *Baie du Marin*, and between 1 and 4 for *Crique du Sphinx*. The string <camera ID-sequence number> is composed of the camera ID that identifies the camera type used for image acquisition, including GPL (GoPro^®^HERO7 LEFT camera), GPR (GoPro^®^HERO7 RIGHT camera), P (Paralenz^®^ Vaquita camera) and B&W (black and white SAAB CAM06P camera); and the sequence ID which is a figure varying from 1 to 12 depending on the transect. The string <year> corresponds to the year of the oceanographic campaign, either 2021 or 2022. Finally, the string <format> is for the format of video sequences, which are all in MPEG-4 (mp4). For example, video imagery BDM-T7_B&W-2_2021 corresponds to transect 7 performed at *Baie du Marin*, sequence 2 of video surveys recorded with the ROV black and white SAAB CAM06P camera during the HA04-Crozet-2021 oceanographic cruise.

### CROCO: CROzet COmmunities data and images

With the exception of BDM-T1x and BDM-T3x transect sections, still images were equally extracted from videos, at a pace of one image every 5 m along the transect, for each survey of the HA04-Crozet-2021 campaign^34^. However, transects conducted during HA04-Crozet-2022 campaign as well as BDM-T1x and BDM-T3x transect sections were totally cut into still images^12^. Images were manually extracted from video-transects using the *VLC media player version 3.0.16* (VideoLAN Organization, Paris, France) software, taking care to avoid overlap and out-of-focus images. Overall, a total of 755 images (670 images for HA04-Crozet-2021; and 85 images for HA04-Crozet-2022) were manually annotated using the online open-source software BIIGLE 2.0 (*Benthic Image Indexing and Graphical Labelling Environment*)^35^. Identification of faunal and algal taxa was performed at the lowest taxonomic level possible in collaboration with taxonomists. This process involved (i) reviewing the images analysed in BIIGLE 2.0 using the *LARGO* tool, which enables a comparison of annotations for a specific taxa, and (ii) submitting collected samples and macro-photography pictures captured during transect imaging acquisition to taxonomists. For each image and each taxon, faunal organisms were counted, and algae surface covers were measured by delineating each one on calibrated images. These biological data were obtained using only one of the two GoPro^®^HERO7 cameras for benthic surveys conducted in shallow waters, and from the colour high-resolution OTAQ Eagle IPZ300™ camera in deeper waters.

**Fig. 4:**
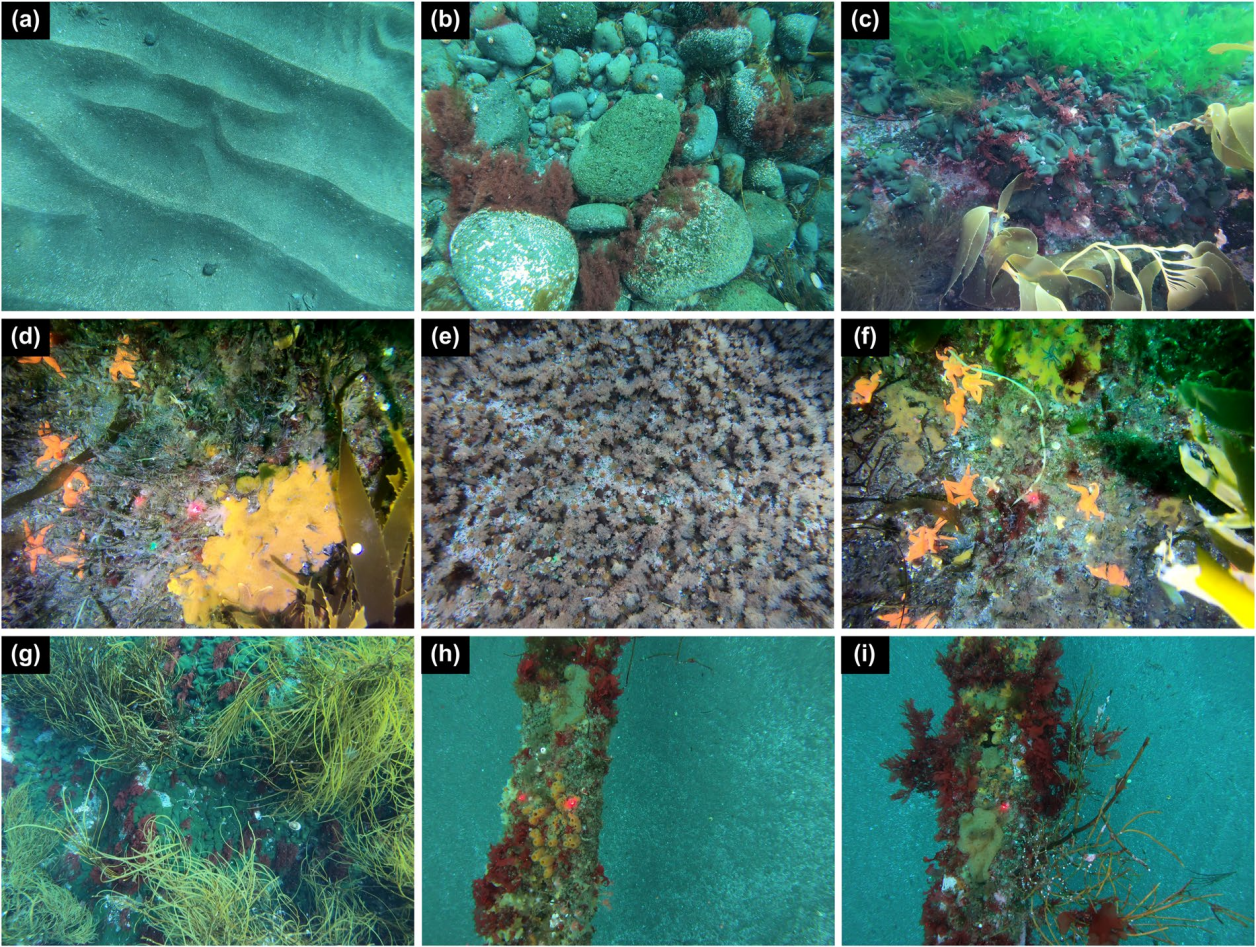
Set of examples of underwater images acquired by the GoPro^®^HERO7 multiple camera set-up in shallow waters of *Baie du Marin* and *Crique du Sphinx*, where algal cover and faunal organisms of each taxa were measured and counted, respectively. Photos highlight different (**a**–**g**) natural and (**h–g**) artificial (submarine cables) habitats.

Within each transect folder, CROCO (Fig. 3b) contains: (i) Benthic Communities Raw Data with two files: faunal abundance data and algal surface cover for each image of the corresponding transect; and (ii) Transect Images used for quantifying faunal abundance and algal surface cover (Fig. 4).

File names in the Benthic Communities Raw Data archive have the following format:

<site-survey ID>_<organism-measurement>_<year>.<format>

Filenames start with the string <site-survey ID> where site is either BDM (*Baie du Marin*) or CdS (*Crique du Sphinx*), and survey ID starts with letter T for “Transect” followed by a figure varying between 1 and 13 for *Baie du Marin*, and between 1 and 4 for *Crique du Sphinx*. The string <organism-measurement> designates the type of organism present in images and associated data (Algal-Cover or Faunal-Abundance data). The string <year> corresponds to the year of the oceanographic campaign, either 2021 or 2022. Finally, the string <format> indicates the file format, either an Excel (.xls) or Comma-Separated Text file (.csv). For example, benthic data CdS-T1_Faunal-Abundance_2021 corresponds to transect 1 performed at *Crique du Sphinx*, faunal abundance counted on analysed images of the transect, during the HA04-Crozet-2021 oceanographic cruise.

Data files have the following, standardized format: image identification, temperature data, depth, image area (computed based on the known distance between laser pointers), habitat type (categorized as sand, sand-cable, sand-pebbles, sand-pebbles-cable, pebbles, suspended-cables, and rock), and a varying number of columns corresponding to either abundance (fauna) or cover (algae) data for each taxa identified along the video-transect. Latitude and longitude of ROV images (BDM-T4 to BDM-T8 as well as CdS-T3 and CdS-T4) are also provided for the HA04 Crozet 2021 survey. Temperature data are missing for ROV transects, but point-based measurements performed in the field indicate a narrow range of 4.8 to 5 °C. With the exception of BDM-T8 and CdS-T3 surveys, algal cover data was not observed along ROV transects.

## Technical Validation

Overall, a total of 755 images were manually analysed, comprising 52 faunal (representing 20,709 annotations) and 14 algal taxa identified in 2021 as well as 45 faunal (representing 28,015 annotations) and 14 algal taxa identified in 2022. Quality of datasets was checked through a detailed analysis of the structural and functional diversity of benthic communities associated with hard substrates using both a part of the HA04-Crozet-2021 dataset and the complete HA04-Crozet-2022 video dataset^12^. Taxonomic identification, species abundance data at the transect scale as well as species functional traits were made available in Lelièvre *et al*.^12^. In addition, a characterisation of coastal habitats and benthic communities was conducted from video-imagery surveys of the HA04-Crozet-2021 dataset^34^. Finally, video surveys from HA04-Crozet-2021 were proved reliable to characterize local fine-scale hydrodynamic processes occurring along the eastern coast of *Ile de la Possession* based on bedforms analysis^36^.

## Usage Notes

### Recommendations for video-imagery manual processing

We suggest using the online open-source platform BIIGLE 2.0 (*Benthic Image Indexing and Graphical Labelling Environment*)^35^ to analyse still images and video-datasets. BIIGLE 2.0 was developed for annotating the benthic fauna from marine image collections using customized tools that make manual annotation efficient and effective^35^. BIIGLE 2.0 offers the following advantages: (i) a user-friendly interface to annotate images or videos with specific labels (e.g., algae cover, species abundance); (ii) an automatic storage of images, labels and annotations on a central server allowing collaborative studies between researchers and taxonomists. According to the invited member status (either guest, editor or admin; access authorization being provided by the administrator user), users may have the permission to add or edit annotations entered in the project; (iii) a standardised data export containing expert labels into a single Excel sheet; and finally, (iv) a high quality technical support that contributes to a positive user experience.

In addition, a few recommendations may be useful to ensure data quality and validity control when manually processing benthic imagery data:

1. To optimize video-imagery annotations and analysis, we advocate an initial “*observer calibration*” training period for users to get familiar with their case study. User knowledge may improve during the annotating process contributing to an increased reliability of observers’ analyses^37^. White *et al*.^38^ suggests that 10% of data should be devoted to the training process, for which we recommend using a random set of video-imagery.
2. To minimize or avoid interrelated observations and maintain a constant level of observer attention over time, we recommend processing images in a random order. Observer fatigue leads to a decrease in cognitive performance during imagery analysis after 60 minutes^39^. To preserve data accuracy, we recommend a 5-minute break every hour.
3. We advise observers to alternately analyse still images and the corresponding video sequences to optimize annotation quality (e.g., species identification, species abundance). In the case of faunal communities studies, several factors including high densities, overlapping between individuals and camouflage by algae may complicate data processing from still images. We thus recommend the use of video sequences, alternately read in backward and forward modes to enhance annotation precision.

### Information on ROV imagery and benthic communities’ data

We recommend users to pay close attention to directional values displayed on the compass of images from the black and white SAAB CAM06P front camera equipped on the ROV (HA04-Crozet-2021). When comparing compass values with the known cable position, it appeared that the compass is out by about 50 degrees. We also inform users about recording issues from 06:04:09 to 06:09:59 on the B&W SAAB CAM06P front camera for transect BDM-T5, and from 12:37:55 to 12:49:33 for the B&W SAAB CAM06P front camera for transect BDM-T6.

Similarly, we would like to notify users about missing data (NA) in three faunal abundance files within the HA04-Crozet-2021 dataset, BDM-T4 (BDM-T4_Image-39_2021, BDM-T4_Image-43_2021 and BDM-T4_Image-46_2021), BDM-T7 (BDM-T7_Image-25_2021 and BDM-T7_Image-26_2021), as well as CdS-T4 (from CdS-T4_Image-51_2021 to CdS-T4_Image-54_2021).

### Data sharing and future benthic researches

The commitment of the French Southern and Antarctic Territories (TAAF) and Biogéosciences Research Unit (CNRS, *Université de Bourgogne*) to share MEDUSA is intended to foster collaborative initiatives in the community of marine ecologists to improve understanding of the functioning of sub-Antarctic ecosystems and address environmental challenges these unique vulnerable environments are confronted with. The provided data are also intended to help decision-makers to shape effective conservation policies for the preservation and sustainable management of these unique environments.

## Data Availability

Faunal abundance and algal surface cover datasets were obtained by using the online software BIIGLE 2.0 (*Benthic Image Indexing and Graphical Labelling Environment*)^35^.

## References

[1] Turner J (2014). Antarctic climate change and the environment: an update. Polar Rec. (Gr. Brit)..

[2] Wang G (2022). Future Southern Ocean warming linked to projected ENSO variability. Nat. Clim. Chang..

[3] Sallée JB (2018). Southern Ocean warming. Oceanography.

[4] Azarian C, Bopp L, Pietri A, Sallée JB, d’Ovidio F (2023). Current and projected patterns of warming and marine heatwaves in the Southern Indian Ocean. Prog. Oceanogr..

[5] Nel W, Hedding DW, Rudolph EM (2023). The sub-Antarctic islands are increasingly warming in the 21st century. Antarct. Sci..

[6] Gille ST (2002). Warming of the Southern Ocean since the 1950s. Science..

[7] Mélice JL, Lutjeharms JRE, Rouault M, Ansorge IJ (2003). Sea-surface temperatures at the sub-Antarctic islands Marion and Gough during the past 50 years. S. Afr. J. Sci..

[8] Auger M, Morrow R, Kestenare E, Sallée JB, Cowley R (2021). Southern Ocean *in-situ* temperature trends over 25 years emerge from interannual variability. Nat. Commun..

[9] McNeil BI, Matear RJ (2008). Southern Ocean acidification: a tipping point at 450-ppm atmospheric CO2. Proc. Natl. Acad. Sci. USA.

[10] Turner J (2009). Non-annular atmospheric circulation change induced by stratospheric ozone depletion and its role in the recent increase of Antarctic sea ice extent. Geophys. Res. Lett..

[11] Blanchard-Wrigglesworth E, Roach LA, Donohoe A, Ding Q (2021). Impact of winds and Southern Ocean SSTs on Antarctic aea ice trends and variability. J. Clim..

[12] Lelièvre Y (2023). Taxonomic and functional diversity of subtidal benthic communities associated with hard substrates at Crozet archipelago (sub-Antarctic, Southern Ocean). Front. Mar. Sci..

[13] von der Meden CEO (2017). Long-term change in epibenthic assemblages at the Prince Edward Islands: a comparison between 1988 and 2013. Polar Biol..

[14] Seitz RD, Wennhage H, Bergström U, Lipcius RN, Ysebaert T (2014). Ecological value of coastal habitats for commercially and ecologically important species. ICES J. Mar. Sci..

[15] Henseler, C. *et al*. Coastal habitats and their importance for the diversity of benthic communities: a species- and trait-based approach. *Estuar. Coast. Shelf Sci*. **226** (2019).

[16] Törnroos A, Nordström MC, Bonsdorff E (2013). Coastal habitats as surrogates for taxonomic, functional and trophic structures of benthic faunal communities. PLoS One.

[17] Hewitt JE, Thrush SF, Dayton PD (2008). Habitat variation, species diversity and ecological functioning in a marine system. J. Exp. Mar. Bio. Ecol..

[18] Friedlander AM (2023). Patterns and drivers of benthic macroinvertebrate assemblages in the kelp forests of southern Patagonia. PLoS One.

[19] Clark GF (2019). Nearshore marine communities at three New Zealand sub-Antarctic islands. Polar Biol..

[20] Freeman D, Cooper S, Funnell G, Neale D (2011). Nearshore benthic community structure at the Bounty and Antipodes Islands, Subantarctic New Zealand. Polar Biol..

[21] Barnes DKA (2006). Shallow benthic fauna communities of South Georgia Island. Polar Biol..

[22] Branch GM, Attwood CG, Gianakouras D, Branch ML (1993). Patterns in the benthic communities on the shelf of the sub-Antarctic Prince Edward Islands. Polar Biol..

[23] Féral, J.-P. *et al*. The marine vegetation of the Kerguelen Islands: history of scientific campaigns, inventory of the flora and first analysis of its biogeographical affinities. *Cryptogam. Algol*. 173–216 (2021).

[24] Teixidó N, Garrabou J, Harmelin JG (2011). Low dynamics, high longevity and persistence of sessile structural species dwelling on mediterranean coralligenous outcrops. PLoS One.

[25] Smale DA, Barnes DKA (2008). Likely responses of the Antarctic benthos to climate-related changes in physical disturbance during the 21st century, based primarily on evidence from the West Antarctic Peninsula region. Ecography (Cop.)..

[26] Teixidó N, Garrabou J, Gutt J, Arntz WE (2004). Recovery in Antarctic benthos after iceberg disturbance: trends in benthic composition, abundance and growth forms. Mar. Ecol. Prog. Ser..

[27] Robinson BJO, Barnes DKA, Grange LJ, Morley SA (2022). The extremes of disturbance reduce functional redundancy: functional trait assessment of the shallow Antarctic benthos. Front. Mar. Sci..

[28] Bravo G (2023). Roving diver survey as a rapid and cost-effective methodology to register species richness in sub-Antarctic kelp forests. Diversity.

[29] Solan M (2003). Towards a greater understanding of pattern, scale and process in marine benthic systems: a picture is worth a thousand worms. J. Exp. Mar. Bio. Ecol..

[30] Jamieson, A. J., Boorman, B. & Jones, D. O. B. Deep-sea benthic sampling. in *Methods for the study of marine benthos* 285–347 (2013).

[31] Bauer, S. & O’Reilly, C. The Comprehensive Nuclear-Test-Ban Treaty Organization (CTBTO): current and future role in the verification regime of the nuclear-test-Ban Treaty. in *Nuclear Non-Proliferation in International Law: Volume II - Verification and Compliance* 131–150 (2016).

[32] Chevallier, L., Nougier, J. & Cantagrel, J. M. Volcanology of Possession island, Crozet archipelago (TAAF). in *Antarctic Earth Science, 4th International Symposium* 652–658 (1983).

[33] Lelièvre Y (2024). dataUBFC.

[34] Lelièvre, Y., Le Gall, L., Dubois, P. & Saucède, T. Characterisation of coastal habitats and marine benthic communities of the sub-Antarctic Crozet archipelago using underwater imagery. *submitted* (2024).

[35] Langenkämper D, Zurowietz M, Schoening T, Nattkemper TW (2017). BIIGLE 2.0 - Browsing and annotating large marine image collections. Front. Mar. Sci..

[36] Lelièvre, Y. *et al*. Can we infer coastal hydrodynamics from bedform patterns and sediment properties? A case study from the sub-Antarctic Crozet archipelago. *submitted* (2024).

[37] Howell, K. L., Bullimore, R. D. & Foster, N. L. *Quality assurance in the identification of deep-sea taxa from video and image analysis: response to Henry and Roberts*. **71**, 899–906 (2014).

[38] White, J. *et al*. Seafloor video mapping: collection, analysis and interpretation of seafloor video footage for the purpose of habitat classification and mapping. MESH Video Working Group Report (2007).

[39] Schoening, T., Osterloff, J. & Nattkemper, T. W. RecoMIA—Recommendations for Marine Image Annotation: lessons learned and future directions. *Front. Mar. Sci*. **3** (2016).

